# Enhancing the Robustness of Smartphone Photoplethysmography: A Signal Quality Index Approach

**DOI:** 10.3390/s20071923

**Published:** 2020-03-30

**Authors:** Ivan Liu, Shiguang Ni, Kaiping Peng

**Affiliations:** 1 Data Science and Information Technology Research Center, Tsinghua-Berkeley Shenzhen Institute, Tsinghua University, Shenzhen 518055, China; liusq15@mails.tsinghua.edu.cn (I.L.); pengkp@tsinghua.edu.cn (K.P.); 2 Graduate School at Shenzhen, Tsinghua University, Shenzhen 518055, China; 3 Department of Psychology, Tsinghua University, Beijing 100084, China

**Keywords:** smartphone photoplethysmography, heart rate variability, signal quality index, pulse waveform

## Abstract

Heart rate variability (HRV) provides essential health information such as the risks of heart attacks and mental disorders. However, inconvenience related to the accurate detection of HRV limits its potential applications. The ubiquitous use of smartphones makes them an excellent choice for regular and portable health monitoring. Following this trend, smartphone photoplethysmography (PPG) has recently garnered prominence; however, the lack of robustness has prevented both researchers and practitioners from embracing this technology. This study aimed to bridge the gap in the literature by developing a novel smartphone PPG quality index (SPQI) that can filter corrupted data. A total of 226 participants joined the study, and results from 1343 samples were used to validate the proposed sinusoidal function-based model. In both the correlation coefficient and Bland–Altman analyses, the agreement between HRV measurements generated by both the smartphone PPG and the reference electrocardiogram improved when data were filtered through the SPQI. Our results support not only the proposed approach but also the general value of using smartphone PPG in HRV analysis.

## 1. Introduction

Heart rate (HR) is an indicator of the balance of multiple physiological systems such as the cerebral cortex, autonomic nervous system, endocrine system, and baroreflex [1,2]. Even while at rest, HR continuously adapts to physiological adjustments such as changes in arterial pressure caused by breathing [3]. By observing HR variability (HRV), researchers can assess our physical capability to adapt to internal physiological requests or changes in our surroundings. 

Studies have linked HRV to several health-related variables such as gender [4], body mass index [5], exercise habits [6], quality of sleep [7,8], insulin resistance [9], and inflammation [10]. A low HRV has been used to predict several health problems including heart attacks [11], headaches [12], and renal impairment [13]. Given that mental states influence the activation of the sympathetic nervous system (SNS) and the parasympathetic nervous system (PNS) [14,15], and that HR is modulated by the SNS and PNS, researchers have associated HRV with mental characteristics such as attention span [14,16], decision making [17], social behavior [18,19], and emotional modulation [20]. 

Despite the importance of HRV in clinical diagnoses and preventative medical applications, the cost and immobility of traditional electrocardiogram (ECG) equipment limits its potential for continuous health monitoring. The advances in information technology have introduced several new approaches to make health information more accessible than before. Among these approaches, smartphone photoplethysmography (PPG) has gained prominence [21,22]; it is an optical method that uses sensors to monitor the microvascular blood volume changes in body tissues [23]. As hemoglobin absorbs more light than the surrounding tissue, an increase (systole) or decrease (diastole) in the amount of blood can be assessed by employing the differences in the intensities of the lights and the converted waveforms. Smartphone PPG detects heartbeats by recording videos of fingertips using the in-built camera [24,25]. 

The primary motivation for using smartphone PPG, compared to other wearable devices, is that it requires minimal equipment. Given that smartphones with in-built cameras have become a part of modern life, using them to access health information is an ideal alternative when ECGs or similar medical devices are not available [26]. In addition, there have been several reported techniques for increasing the accuracy of smartphone PPG, such as point-of-interest selection [27], bandpass filtering [28], adaptive signal thresholding [29], motion detection techniques [30,31,32], interpolation techniques [33], and signal decomposition methods [34,35,36]. Bioengineering studies indicate that the average HR [37] and HRV measured using smartphone PPG are comparable with those measured using gold standard ECGs [21,28,38,39,40].

Although it is a promising solution for practical data collection and has an accuracy that has been well proved in several experiments, using smartphone PPG to measure HRV has received limited research attention in applied disciplines such as medicine or psychology [41]; a possible explanation is the lack of robustness in practical scenarios. When smartphone users measure their heartbeats outside a laboratory environment, slight hand movements or ambient light changes can corrupt the PPG signals. Software designed to process the camera signals have limited control over the underlying operating system and hardware. Further, camera settings—especially exposure and white balance—differ between smartphone models and may change automatically when the environment changes. The low frame (sampling) rate of smartphone cameras is another source of randomness [42,43]. The sampling rate can be as high as 1000 Hz for medical equipment [44]; however, for most smartphone cameras, it is less than 30 Hz [40], and this can result in the fiducial point detection technique (FPDT) easily missing the actual point. Frame rate instability—as a design to prevent the CPU from overloading or overheating—further degrades the acquisition performance [26,45].

While it is difficult to ensure the robustness of using smartphone PPG in assessing HRV against a less controlled environment, other strategies can be employed to make smartphone PPG practically workable. Users today are familiar with various consumer-grade healthcare devices such as home blood pressure monitors or ECG chest straps. Several of these devices, while validated in laboratories, also have limited mechanisms to deal with randomness found in real-life scenarios. A common strategy to overcome such limitations is to provide measurement quality information so that users can discard corrupted data and reperform the measurement. However, only a few smartphone PPG studies have considered this potential solution [46]. 

This study aimed to bridge the gap in the literature by providing a smartphone PPG quality index (SPQI) that can filter out low-quality data and perform HRV measurements that are comparable to the results from ECGs. We propose a novel approach to fit collected data points to a pre-defined model and calculate the success rate as an index of signal quality. Based on the physiological studies of the radial pulse waveform [47], the current study designed a sinusoidal function-based regression model that can fit the right-skewed pulse signal and determine the quality of data using the success rate of the convergence in the optimization. Further, we conducted an empirical experiment to validate the proposed approach. The potential uses and limitations are also discussed before concluding this paper.

## 2. Materials and Methods 

### 2.1. Signal Pre-Processing

The current study employed three steps to convert film frames of the fingertip to pulse waveforms for further analysis.

#### 2.1.1. Signal Extraction and Conversion

**Figure 1:**
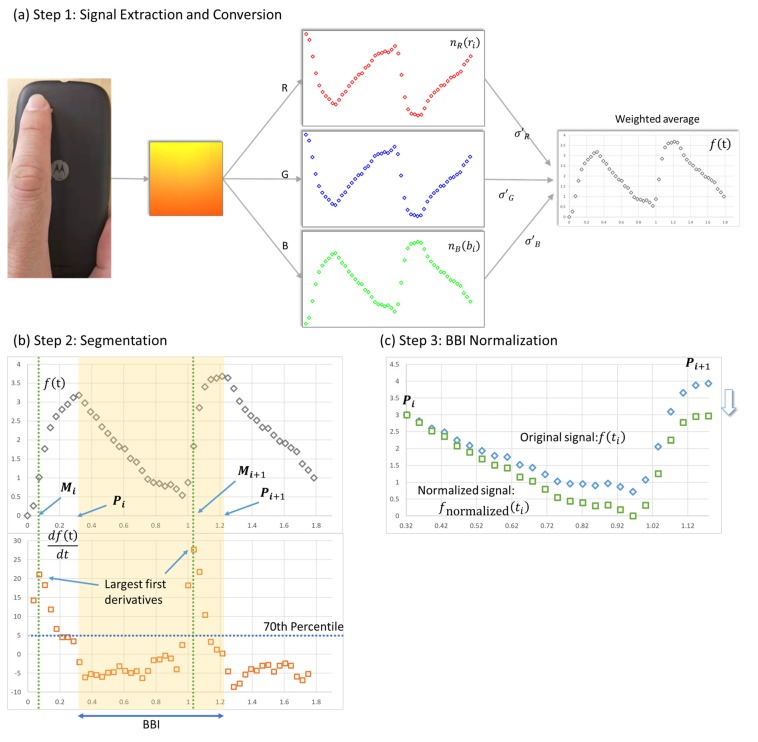
Steps taken to convert raw signals to segmented and normalized pulse waveform.

For each data collection session, a self-developed app first activated the in-built flashlight and recorded 120 × 160 pixel videos with approximately 30 frames per second (the actual frame rate was determined by the underlying operating system) (see Figure 1a). Raw YUV-format picture frames retrieved from the preview function were then converted into the RGB format. Then, the input signals $\left(r_{i},b_{i},g_{i}\right)$ from the three color channels (red, blue, and green) were normalized using the 100-point moving average ($\bar{R},\;\bar{B},\;\bar{G}$) and standard deviation ($\sigma_{R},\;\sigma_{B},\;\sigma_{G}$):

(1) $$n_{R}\left(r_{i}\right)=\frac{r_{i}-\bar{R}}{\sigma_{R}},n_{B}\left(b_{i}\right)=\frac{b_{i}-\bar{B}}{\sigma_{B}},n_{B}\left(g_{i}\right)=\frac{g_{i}-\bar{G}}{\sigma_{G}},\;i\in A=\left(1,\ldots ,k\right),$$

where k is the amount of data collected in each data collection session. Because standard deviation can represent the relative strength of each channel, the signals were combined with the standard deviation-weighted average as follows:

(2) $$\;f\left(t_{i}\right)=\frac{\sigma \prime_{G}\times n_{G}\left(g_{i}\right)-\sigma \prime_{B}\times n_{B}\left(b_{i}\right)-\sigma \prime_{R}\times n_{R}\left(r_{i}\right)}{\sigma \prime_{R}+\sigma \prime_{G}+\sigma \prime_{B}},$$

where

$$\sigma_{C}^{\prime }=\{\begin{array}{cc} \sigma_{C} & \mathrm{if}\;\sigma_{C}>0.5,\;3<\bar{C}<252\;\\ 0 & \mathrm{otherwise}\; \end{array},\;C\in \left\{R,B,G\right\}$$

and $t_{i}\in T=\left\{t_{i}|i\in 1,..,k\right\}$ denotes the time at which the i^th^ data point was collected. A color channel was removed from the weighted average $f\left(t_{i}\right)$ when the average of the input ($\bar{C}$) was either too small or too close to the upper limit of 255, or when the standard deviation ($\sigma_{C}$) was too small ($\sigma_{C}\leq 0.5$). In addition, the signs of the red and blue channel were reversed to denote the inverse relationship between the green channel and the other two channels.

#### 2.1.2. Beat-to-Beat Interval (BBI) Segmentation

After converting the signals to a waveform input, we divided the “continuous” waveform dataset $f\left(t_{i}\right)$ into segments representing each beat-to-beat interval (BBI). Given that the radial pulse waveform follows a right-skewed bell shape, distinct plateaus can be observed on the first derivatives during each heartbeat (see Figure 1b). Although the exact position of the maximum of the waveform is susceptible to noise, the detection of the plateau is relatively robust. Therefore, this study used a set of local maxima ($M_{i}$)—above the 70th percentile ($P_{70}$) of the first derivatives $f^{\prime }\left(t_{i}\right)$—to identify potential BBIs for further analysis.

(3) $$M=\left\{M_{1},M_{2},\ldots ,M_{u}\right\}=\left\{t_{i}|\max f^{\prime }\left(t_{i}\right)>P_{70},\;f^{\prime \prime }\left(t_{i}\right)<0,\;\frac{60}{t_{j}-t_{i}}<150\;\forall j\neq i,\;t_{i}\in T\right\}.$$

The distances between two successive data points in M were converted into HRs to filter out points with HRs over 150. All first and second derivatives were calculated using first- and second-order central difference approximations. The set of maximum points ($P_{i}$) of the waveform between two data points in M is defined as P:

(4) $$P=\left\{P_{1},P_{2},\ldots ,P_{q}\right\}=\left\{t_{j}|m_{j}<t_{j}<m_{j+1},\;f\left(t_{j}\right)=\max\left\{f\left(t_{i}\right)|m_{j}\leq t_{i}<m_{j+1}\right\}\right\}.$$

The intervals segmented by the data points in P are then defined as the set of BBIs (B):

(5) $$B=\left\{\left(t_{1},t_{2}\right)|t_{1}\in P,t_{2}=\min\left\{t_{i}\in P\;|t_{i}>t_{1},\frac{60}{t_{i}-t_{1}}<150\right\}\right\}.$$

#### 2.1.3. BBI Normalization

To reduce the problem caused by baseline drifting [40], the amplitudes of data points in each BBI were normalized in proportion to the height difference of the two successive peaks as described below (see Figure 1c):

(6) $$f_{\mathrm{normalized}}\left(t_{i}\right)=f\left(t_{i}\right)+\left(\frac{t_{i}-t_{L}}{t_{R}-t_{L}}\right)\times \left(f\left(t_{R}\right)-f\left(t_{L}\right)\right),\;\forall t_{i}\in \;\left(t_{L},t_{R}\right),\;\left(t_{L},t_{R}\right)\in B\;$$

The normalized data points were used to fit the regression model and to determine heartbeat points with various FPDTs. The current study then applied the R package RHRV [48] to convert the heartbeat points generated by the FPDTs to HRV measures for further analysis.

### 2.2. Sinusoidal Function-Based Photoplethysmography (PPG) Quality Index

Although the exact contour of the waveform is influenced by the characteristics of the individuals’ systemic circulation [49], the general shapes are similar. For most healthy people, the contour is right skewed and bell shaped, with a dicrotic notch in the middle [50]. Since the shapes of the pulse waveforms are similar, PPG studies use waveform morphology to differentiate acceptable signals from contaminated ones [51]. 

Two families of functions have been used to describe the pulse waveform: Gaussian (or modifications, such as the Rayleigh functions) [46,52,53,54] and sinusoidal functions. Since sinusoidal functions are more commonly used in hemodynamic studies (i.e., Fourier analysis) to predict health-related variables [55], and are less computationally demanding, this study fit the pulse waveform with the sum of sinusoidal functions. 

**Figure 2:**
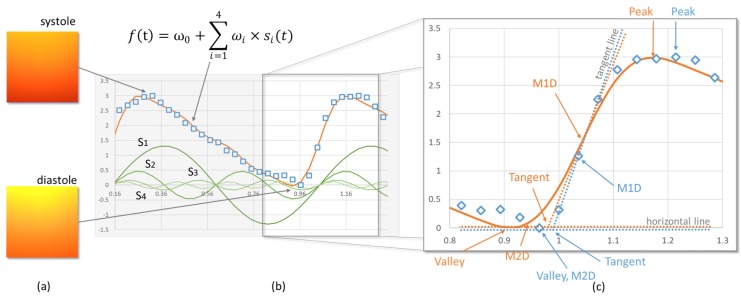
(**a**) Illustration of two frames of the video of a left fingertip captured using a smartphone camera (Mi 8 SE, Xiaomi, China). The frame captured during the systole phase is darker and converted to a larger value by photoplethysmography (PPG); the frame captured during the diastole phase is lighter and converted to a smaller value by PPG. (**b**) Illustration of using the sum of the four sinusoidal functions to fit the collected samples from a 19-year-old female. (**c**) Illustration of five types of fiducial points (Peak, Valley, Tangent, maximum first derivative (M1D), and maximum secondary derivative (M2D)) determined with both raw data points (blue) and the fitted model (orange).

We define the model as:

(7) $$f\left(t\right)=\omega_{0}+\overset{n}{\underset{i=1}{\sum }}\omega_{i}\times s_{i}\left(t\right)\text{ The\_SMF}$$

where $s_{i}\left(t\right)=\sin\left(i\times c\times \left(t-h\right)\times \pi \right)$ is the i^th^ sinusoidal function, $\omega_{i}\geq 0$ is the weighting of the i^th^ sinusoidal function, c∈R is the scaling parameter, h∈R is the displacement parameter, and n is the number of sinusoidal functions included in the model (Figure 2a,b). As an explorative study, the frequencies of the sinusoidal functions ($s_{i}\left(t\right),i>1$) were provided as multipliers of the base function $s_{1}\left(t\right)$ to reduce model complexity. In addition, the weightings $\omega_{i}$ were restricted to positive values and $w_{1}\geq 2\times w_{i}\;\forall i>1$ to keep the model (Equation (1)) right skewed and approximately bell shaped. Future studies may relax these constraints.

The model fit the input data points using nonlinear least-squares optimization with 10,000 iterations. Based on previous experience, we used a set of initial values: $\left(w_{0},w_{1},w_{2},w_{3},w_{4},c,h\right)=\left(7,7,3,1,1,2,0.1\right)$. Since the sum of the squared errors in the regression did not change significantly when *n* was greater than four in our preliminary data analysis, we set *n* = 4 in the model. When the waveform of an interval was severely corrupted, or the section contained many artifacts, the model failed to converge before reaching the maximum number of iterations or had a large root mean square error (RMSE). The model fitting was considered to have failed when the RMSE was larger than 0.5. The maximum number of iterations and the threshold for the RMSE were determined based on experience; future studies may re-examine these constraints. Further, because the pulse waveform was relatively stable, for each BBI, we used the fitting results from previous BBIs to filter out parameters that were outliers. When either $\omega_{1}$ and $\omega_{2}$ were outside of the boundary determined by Tukey’s method, i.e., more than 1.5 times the interquartile range beyond the quartiles, the fitting was considered to have failed. We then defined the SPQI as the success rate of the model-fitting process.

(8) $$\mathrm{SPQI}=\frac{\mathrm{number}\;\mathrm{of}\;\mathrm{successful}\;\mathrm{model}\;\mathrm{fittings}}{\mathrm{number}\;\mathrm{of}\;\mathrm{BBIs}}\text{ The\_SMF}$$

### 2.3. HRV Measures

There are three types of HRV measures: (1) time-domain, (2) frequency-domain, and (3) nonlinear. The frequency-domain components of HRV consist of four frequency bands: high frequency (HF), low frequency (LF), very low frequency (VLF), and ultralow frequency (ULF) (Table 1). Given that this study only recorded 5-min videos, the ULF and VLF bands did not apply [56]. The HF and LF values were log-transformed because they are not distributed normally [43,57]. The time-domain indices of HRV quantify variability in the BBI. This study included three commonly used time-domain measures for comparison: rMSSD, pNN50, and SDNN. Nonlinear HRV measures are computationally complex and were accordingly excluded from this study.

### 2.4. FPDT

The current study included five frequently used FPDTs to compare the agreement between smartphone PPG and the reference ECG [26,29,58,59,60,61,62,63,64] (Table 2 and Figure 2c).

### 2.5. Agreement Analysis

We used two methods to compare the agreement between the smartphone PPG and reference ECG. First, we examined the Pearson correlation coefficients of the data generated by the smartphone PPG to the reference ECG. The correlation coefficients (r) were assessed with the Student’s *t*-test where

(9) $$t\;\mathrm{value}=r\sqrt{\frac{n-2}{1-r^{2}}}\text{ Combined\_Signal}$$

Second, we compared the agreement with the Bland–Altman method [65]. The Bland–Altman ratio (BAR) is defined as:

(10) $$\mathrm{BAR}=\frac{0.5\times \left[\max\left(\mathrm{LA}\right)-\min\left(\mathrm{LA}\right)\right]\;}{\mathrm{MPM}}\text{ Combined\_Signal}$$

where LA is the half range of agreement limits (± 1.96 × SD), and MPM denotes the mean of the pairwise mean. The two measurements are considered to have a good or acceptable agreement when the BAR is less than 10% or 20% [40,66].

### 2.6. Participants and Data Collection

The study protocol was approved by the Ethical Board of the Department of Psychology, Tsinghua University; 226 students and university employees in Shenzhen, China, joined the study. The average age was 23.4 years (σ = 3.36) with equal percentages of male and female participants. After a 5-min debriefing, participants were asked to remain seated for the entire data collection process. They were asked to wear an ECG chest strap (H10, Polar Electro Oy, Finland; sampling rate 1000 Hz [67]) and hold a smartphone (Mi 8 SE, Xiaomi, China; sampling rate 30 Hz) in their left hand. A self-developed app was then used to record 5-min videos of their fingertip multiple times during the 1-h session. A total of 1343 valid datasets were collected. The accuracy of the ECG chest strap is well-established in the literature [21], and studies employ the chest strap in detecting HRV for convenience when a 24-lead ECG is not available [68,69].

## 3. Results

### 3.1. Correlation Coefficient Analysis

Before starting data analysis, this study applied Tukey’s rule to remove outliers. All remaining HRV measurements using smartphone PPG were significantly correlated (p < 0.05) with the results detected using ECG (Table 3). In general, smartphone PPG provided better estimations for log HF (across the FPDTs, average r=0.72), log LF (average r=0.70), and SDNN (average r=0.73) than for rMSSD and pNN50. Among the FPDTs, Tangent produced the best results, followed by Valley and M1D; however, M2D had the poorest performance. When the data were filtered with the SPQI, the correlation coefficients increased significantly. On average, they increased by 13% (from 0.669 to 0.758) and 26% (from 0.669 to 0.843) when the data were filtered with SPQI thresholds above 0.8 and 0.95, respectively. The improvement was particularly prominent for M2D, as the average correlation coefficient improved by 51% (from 0.472 to 0.712).

**Figure 3:**
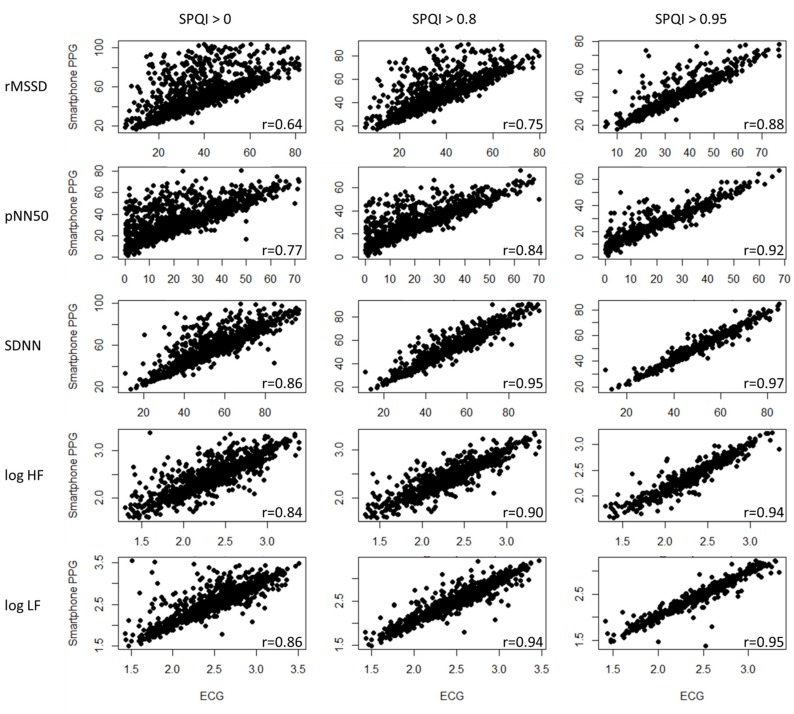
Scatter plot and correlation coefficients of smartphone PPG (with Tangent) and the reference ECG for the HRV measurements.

The advantage of using the SPQI is also apparent from the scatter plots (Figure 3). When all data are included, a significant portion lies away from the straight line; when corrupted data are filtered out, a higher portion of the data lies along the straight line. In particular, there is an asymmetric bias for rMSSD and pNN50. Smartphone PPG tended to produce larger values for these measures when the signal quality was low, and therefore, their correlation coefficients were lower. Since rMSSD and pNN50 are more susceptible to the randomness of the samples, the benefits of using the SPQI were also more prominent for these two measures. When we filtered the data with an SPQI threshold level of 0.95, the correlation coefficients for rMSSD and pNN50 increased from 0.64 and 0.77 to 0.88 and 0.92, respectively.

However, when the data set was filtered with an SPQI > 0.95, the reduction in the number of samples was not negligible (see Table 4). On average, the number of samples decreased by 14% (from 1263 to 1060) and 56% (from 1263 to 557) when the data were filtered out by thresholds above 0.8 and 0.95, respectively.

### 3.2. Bland–Altman Ratio Analysis

The Bland–Altman analysis showed similar results to the correlation coefficient analysis. Among all FPDTs, Tangent generated the smallest BAR for SDNN, log HF, and log LF (see Table 5). M1D, in contrast, performed the best for rMSSD, and Valley had the lowest BAR for pNN50. The agreement of log HF and log LF was “acceptable” (BAR < 0.2) before filtering with the SPQI. The agreement of log HF and log LF generated by all FPDTs became “good” or close to “good” when the data were filtered with an SPQI > 0.95.

**Figure 4:**
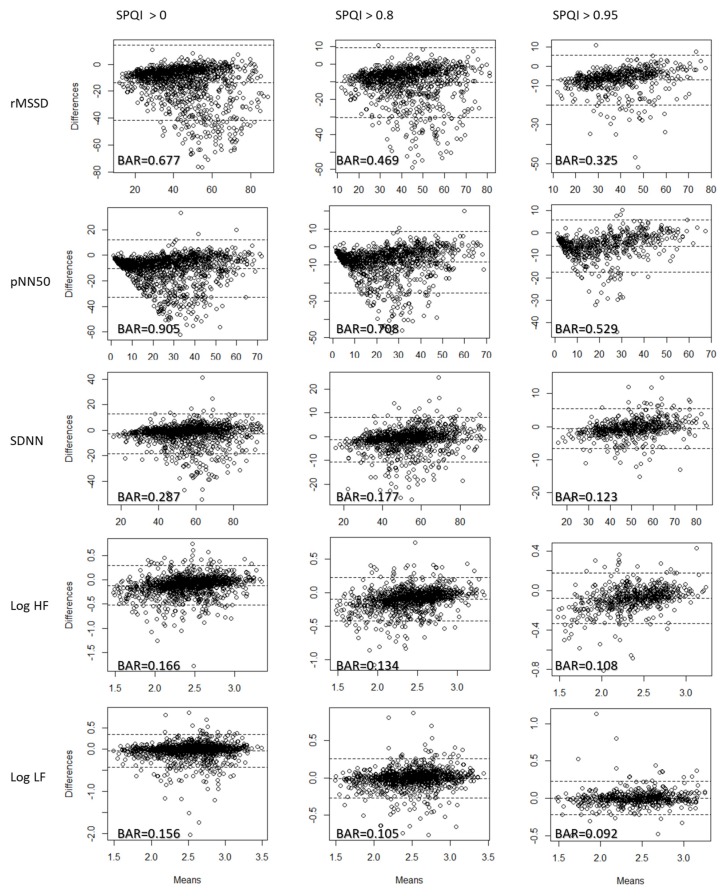
Bland–Altman plot and BAR smartphone PPG (with Tangent) and reference ECG data for each HRV measure.

The effect of the SPQI can also be observed from the Bland–Altman plot (Figure 4). Considering data generated by Tangent, the number of points that lie beyond the upper and lower agreement limits was significantly reduced when the data were filtered with the SPQI. The same pattern was observed for all HRV measures. Similar to the correlation coefficient analysis, rMSSD and pNN50 showed the least agreement among HRV measures. Although filtering with an SPQI > 0.95 could significantly reduce the BAR, these two measures were still above the “acceptable” level for all FPDTs.

## 4. Discussion

### 4.1. Principal Findings

The results from both the correlation coefficient and Bland–Altman analyses validated our proposed strategy: removing data that cannot fit a pre-defined model can significantly increase the accuracy of smartphone PPG. By choosing a relatively more robust FPDT, such as Valley or Tangent, and filtering data with the SPQI, the agreement between smartphone PPG and ECG can have a “good” agreement, and the correlation coefficient can be over 0.9.

Among the FPDTs, Tangent and M2D were generally the best performers [40,62]. Our data showed that Tangent had the best agreement with ECG for SDNN, log HF, and log LF, while M1D and Valley had the highest agreement for rMSSD and pNN50, respectively; M2D was the worst performer in our data. Given that M2D performed well in previous studies, our results suggest that M2D may be more sensitive to the randomness found in corrupted data (which was manually removed in previous studies).

Compared with rMSSD, pNN50, and HF, SDNN and LF generated by smartphone PPG generally have a higher agreement with the reference ECG [33,40]; our data demonstrated similar results. Neither rMSSD nor pNN50 reached an acceptable agreement between the smartphone PPG and the ECG, even with SPQI > 0.95 filters. Our data showed that these two measures were more susceptible to randomness and systematic bias. SDNN and log LF had an acceptable or good agreement when the data were generated by Valley, M1D, or Tangent. The same was true for log HF.

### 4.2. Limitations

Our data support the proposed method; however, there are still some limitations, as listed below. 

First, the underlying assumption of the SPQI is that most people share common cardiac waveform characteristics. Although this hypothesis is based on empirical studies [47], this premise limits the application of the SPQI to individuals with abnormal cardiac waveforms since the SPQI classifies samples that do not meet the preset pattern as poor quality. We expect future studies may pursue this research direction and try to differentiate corrupted data from valid but abnormal samples.

Second, the assumed application scenario of the proposed method is to determine the quality of a sample collected from a new participant based on the theoretical waveform pattern found by previous studies. We did not consider the possibility of using historical data for each individual to build a personalized quality index. Since cardiac waveforms have a larger between-group deviation (compared to other people) and smaller within-group differences (compared to one’s historical data), a personalized quality index may help increase the accuracy, and resolve the shortcoming that the SPQI is not suitable for individuals with abnormal waveforms.

Third, there are many methods for improving smartphone PPG accuracy [27,29,30,31,32,33]. For example, adding a suitable bandpass filter for signal processing [28] or excluding data with RR intervals that differ more than a certain threshold [70] are simple and effective approaches to reduce noise. In the current study, however, we did not use other proven noise reduction methods because we aimed to compare the relative accuracy of data filtered with the quality index rather than increase absolute accuracy. Whether the combination of the noise reduction methods and a quality index can further increase the accuracy and usability of smartphone PPG still warrants further analysis.

Fourth, the sampling rate has a significant influence on the accuracy of HRV measures. Although there have been many different suggestions for the minimum sampling rate (ranging from 25 Hz [42] to 125 Hz [43] in PPG studies and 50 Hz [71] to 1000 Hz [72] in ECG studies), most smartphone cameras sample at about 30 Hz, which is below the level of most suggestions. Therefore, the poor measuring quality caused by low frame rates is generally considered a potential challenge to the validity of using the smartphone PPG method. However, researchers have proposed various methods to improve the accuracy rate at low frame rates [33], and empirical studies have also indicated that smartphone PPG results are comparable to those obtained using gold standard ECGs [21,28,37,38,39,40]. There seems to be conflicting suggestions and conclusions in the literature, and therefore, more empirical evidence is required to clarify this issue. Further, smartphone-based physiological assessment applications are usually considered low-cost, convenient tools for public health and personal use. Many smartphone PPG studies, including the current study, aim to validate this new technology as an acceptable alternative when more sophisticated devices are not available, rather than using it as a substitute for medical-grade equipment.

Fifth, several traditional PPG quality indicators have been proposed in the literature [51,73]. However, the design of the smartphone is different from traditional medical PPG devices, which usually have a higher sampling rate [74], are designed to reduce motion artifacts, and use transmitted light sources rather than reflected light sources. Besides, most traditional PPG quality indicators were not validated with HRV measures [73]. It is still unclear whether these traditional PPG quality indicators are applicable to smartphone PPG data and HRV assessment.

Sixth, in this exploratory study, we proposed only one model design and did not compare other possible alternatives such as using the Gaussian function family or changing optimization constraints. Future studies may consider conducting an optimal parameter search and finding better model designs for the SPQI. 

**Figure 5:**
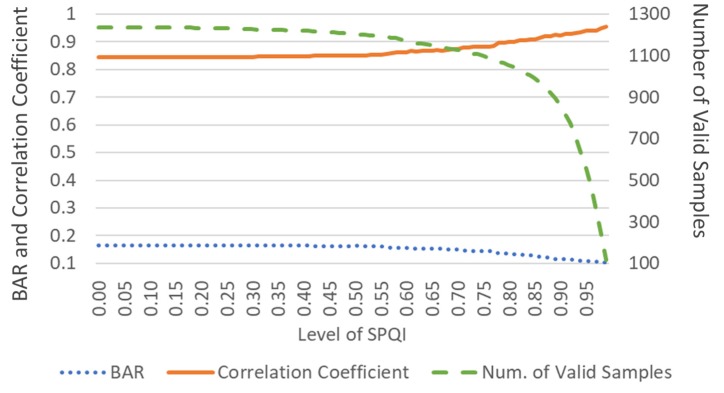
Trade-off between the number of valid samples and the agreement (BAR or correlation coefficient) of log HF between the smartphone PPG (Tangent) and reference ECG.

Finally, the higher the threshold, the higher the accuracy, and the fewer the valid samples (Figure 5). The balance of the measurement quality and external validity of the research results is an essential requirement that should be considered carefully in future studies.

## 5. Conclusions

Smartphone PPG provides an unprecedented opportunity for both researchers and practitioners in clinical diagnoses, telemedicine, preventative medicine, and public health. Research on smartphone PPG also provides a theoretical foundation for several new research directions such as remote photoplethysmography [75] and PPG-based blood pressure estimation [76]. Although it may not become a substitute for the gold standard ECG, smartphones are easily accessible and reasonably accurate alternatives when medical-grade devices are extremely costly or unavailable (e.g., when dealing with an unexpected large-scale public health crisis, such as the recent coronavirus outbreak [77,78]). It is, however, an unfortunate reality that only a few researchers and ordinary users have used this new technology. The proposed quality index enables users to assess the credibility of the gathered HRV measures, which is essential to win the trust of practitioners or researchers in applied disciplines.

The number of participants in this study (n = 226 participants and 1336 collected samples) was relatively large compared with several other smartphone PPG studies [21,38,39,40,79]. Therefore, the results from this study provide support, not only for the validity of the proposed SPQI, but also for the general value and practicality of using smartphone PPG in HRV analysis.

## Figures and Tables

**Table 1:** Definitions of time-domain and frequency-domain heart rate variability (HRV) measurements.

HRV Measures	Definition
**Time-Domain**	
**SDNN**	Standard deviation of the average normal-to-normal (NN) intervals
**pNN50**	Percentage of successive NN intervals that differ by more than 50 ms
**rMSSD**	Root mean square of successive NN interval differences
**Frequency-Domain**	
**HF**	Absolute power of the high-frequency band (0.15–0.4 Hz)
**LF**	Absolute power of the low-frequency band (0.04–0.15 Hz)
**VLF**	Absolute power of the very-low frequency band (0.003–0.04 Hz)
**ULF**	Absolute power of the ultra-low frequency band (≤0.003 Hz)
**log HF**	Log-transformed HF
**log LF**	Log-transformed LF
**log LF/HF**	Log-transformed ratio of LF to HF

**Table 2:** Definitions of fiducial point detection techniques (FPDTs).

FPDT	Fiducial Point Definition
**Peak**	The maximum point in each BBI.
**Valley**	The minimum point in each BBI.
**M1D**	The maximum point of the first derivative in each BBI.
**M2D**	The maximum point of the second derivative in each BBI.
**Tangent**	The point where the tangent line from the M1D intersects the horizontal line from the Valley. The first derivatives of a discrete data set are determined by the difference function approximation.

**Table 3:** Correlation coefficients between the results generated by smartphone PPG and ECG.

Threshold	FPDT	rMSSD	pNN50	SDNN	log HF	log LF	Avg. (FPDT)	Avg. (SPQI)
**SPQI > 0**	Peak	0.520	0.652	0.692	0.639	0.607	0.622	0.669
	Valley	0.608	0.731	0.791	0.758	0.741	0.726	
	M1D	0.596	0.675	0.823	0.790	0.807	0.738	
	M2D	0.290	0.559	0.489	0.549	0.475	0.472	
	Tangent	0.615	0.752	0.864	0.843	0.858	0.786	
**SPQI > 0.8**	Peak	0.604	0.715	0.786	0.699	0.678	0.696	0.758
	Valley	0.702	0.834	0.879	0.844	0.842	0.820	
	M1D	0.705	0.777	0.915	0.846	0.882	0.825	
	M2D	0.393	0.665	0.626	0.629	0.536	0.570	
	Tangent	0.756	0.848	0.947	0.900	0.936	0.877	
**SPQI > 0.95**	Peak	0.689	0.768	0.847	0.799	0.749	0.770	0.843
	Valley	0.898	0.911	0.967	0.914	0.934	0.925	
	M1D	0.795	0.851	0.943	0.881	0.892	0.872	
	M2D	0.565	0.800	0.802	0.762	0.632	0.712	
	Tangent	0.879	0.923	0.974	0.939	0.954	0.934	
**All correlation coefficients in the table have *p* < 0.05**								

**Table 4:** Number of valid samples filtered by different smartphone PPG quality index (SPQI) levels.

Threshold	FPDT	rMSSD	pNN50	SDNN	log HF	log LF	Avg. (FPDT)	Avg. (SPQI)
**SPQI > 0**	Peak	1283	1331	1276	1226	1257	1274.6	1263
	Valley	1258	1329	1269	1245	1255	1271.2	
	M1D	1233	1330	1276	1250	1262	1270.2	
	M2D	1227	1321	1204	1189	1223	1232.8	
	Tangent	1250	1325	1274	1236	1246	1266.2	
**SPQI > 0.8**	Peak	1067	1087	1075	1049	1071	1069.8	1060
	Valley	1062	1083	1073	1056	1066	1068.0	
	M1D	1052	1085	1067	1054	1068	1065.2	
	M2D	1006	1078	1012	1032	1057	1037.0	
	Tangent	1046	1081	1065	1053	1064	1061.8	
**SPQI > 0.95**	Peak	565	550	566	558	565	560.8	557
	Valley	563	548	567	562	562	560.4	
	M1D	561	545	564	555	560	557.0	
	M2D	542	546	553	548	560	549.8	
	Tangent	562	548	564	557	563	558.8	

**Table 5:** Bland–Altman ratios between the results generated by smartphone PPG and ECG.

Threshold	FPDT	rMSSD	pNN50	SDNN	Log HF	Log LF
**SPQI > 0**	Peak	0.694	0.888	0.443	0.232	0.268
	Valley	0.552	0.813	0.344	0.199 *	0.215
	M1D	0.539	0.833	0.312	0.186 *	0.181 *
	M2D	0.848	0.940	0.621	0.256	0.315
	Tangent	0.677	0.905	0.287	0.166 *	0.156 *
**SPQI > 0.8**	Peak	0.581	0.798	0.351	0.214	0.241
	Valley	0.451	0.660	0.262	0.164 *	0.165 *
	M1D	0.433	0.701	0.220	0.164 *	0.141 *
	M2D	0.653	0.808	0.455	0.233	0.291
	Tangent	0.469	0.708	0.177 *	0.134 *	0.105 *
**SPQI > 0.95**	Peak	0.514	0.737	0.283	0.180 *	0.217
	Valley	0.297	0.547	0.144 *	0.129 *	0.108 *
	M1D	0.367	0.615	0.179 *	0.150 *	0.136 *
	M2D	0.504	0.672	0.308	0.195 *	0.257
	Tangent	0.325	0.529	0.123 *	0.108 *	0.092 *
* acceptable or good agreement						

## Supplementary Materials

The following are available online at https://www.mdpi.com/1424-8220/20/7/1923/s1.
